# Head-nodding: a driving force for the circulation of cerebrospinal fluid

**DOI:** 10.1038/s41598-021-93767-8

**Published:** 2021-07-09

**Authors:** Qiang Xu, Chang-Xi Shao, Ying Zhang, Yu Zhang, Cong Liu, Yu-Xiao Chen, Xue-Mei Wang, Yan-Yan Chi, Sheng-Bo Yu, Hong-Jin Sui

**Affiliations:** 1 Department of Anatomy, Dalian Medical University, Dalian, 116044 China; 2 Department of Radiology, The 967 Hospital of the Joint Logistics Support Force of PLA, Dalian, 116021 China; 3 Department of Anesthesiology, Baishan Municipal Central Hospital, Baishan, 134300 China; 4 Graduate School, Dalian Medical University, Dalian, 116044 China; 5 Department of Radiology, Dalian Municipal Central Hospital, Dalian, 116033 China

**Keywords:** Neuroscience, Anatomy

## Abstract

The myodural bridge (MDB) is a dense connective tissue bridge connecting the suboccipital muscles to the spinal dura mater, and it has been proven to be a normal common existing structure in humans and mammals. Some scholars believe that the suboccipital muscles can serve as a dynamic cerebrospinal fluid (CSF) pump via the MDB, and they found head rotations promote the CSF flow in human body, which provided evidence for this hypothesis. Head movement is a complex motion, but the effects of other forms of head movement on CSF circulation are less known. The present study explored the effects of head-nodding on CSF circulation. The CSF flow of 60 healthy volunteers was analyzed via cine phase-contrast magnetic resonance imaging at the level of the occipitocervical junction before and after one-minute-head-nodding period. Furthermore, the CSF pressures of 100 volunteers were measured via lumbar puncture before and after 5 times head-nodding during their anesthetizing for surgical preparation. As a result, it was found that the maximum and average CSF flow rates at the level of the upper border of atlas during ventricular diastole were significantly decreased from 1.965 ± 0.531 to 1.839 ± 0.460 ml/s and from 0.702 ± 0.253 to 0.606 ± 0.228 ml/s respectively. In the meantime, the changes in the ratio of cranial and caudal orientation of the net flow volume were found differed significantly after the one-minute-head-nodding period (p = 0.017). And on the other hand, the CSF pressures at the L3–L4 level were markedly increased 116.03 ± 26.13 to 124.64 ± 26.18 mmH_2_O. In conclusion, the head-nodding has obvious effects on CSF circulation and head movement is one of the important drivers of cerebrospinal fluid circulation. We propose that the suboccipital muscles, participating in various head movements, might pull the dura sac via the myodural bridge, and thus, head movement provides power for the CSF circulation.

## Introduction

The MDB is a kind of dense connective tissue bridge which connects spinal dura mater to suboccipital muscles and ligaments^1–6^. Recently, the existence of the MDB has been revealed in five mammalian species and it is presumed that the MDB commonly exists in mammals^7–9^. The MDB-like structures were also found in the common rock pigeon (*Columba livia*)^10^, Gallus domesticus^11^ and Crocodile (*Crocodylus siamensis*)^12^. This shows that the MDB is a highly conserved evolutionary structure. In general, a universal and highly conserved structure always has its own unique functions.

Some scholars^1,13–17^ speculated that the MDB could prevent dura mater infolding and maintain normal CSF circulation. Other scholars^18^ believed that changes of dural tension could be transmitted through the sensation, triggering contraction or relaxation of the suboccipital muscles. Recent studies^6,19^ proposed that the MDB may act as a dynamic pump for CSF circulation. A study confirmed that head rotations could affect the CSF flow rates^20^. During the head-rotation, the suboccipital muscles could pull the upper cervical dura sac via their MDBs acting as a pump to provide dynamics for the circulation of the CSF^20^.

Head movements are complicated, and varied, while except for head rotation, the effects of other forms of head movement on CSF circulation are less well known. In order to verify relationships between the CSF circulation dynamics and head movements, and to provide physiological data for revealing the role of MDB, the present study explored the effects of head-nodding movement on CSF circulations.

## Materials and methods

Approval from the Ethics Committee for Research at the Basic Medical College of Dalian Medical University was obtained for this study.

### Cine-PC MR imaging measurement of CSF circulation

#### Subject

The experimental protocol of this study was carried out in accordance with the approved guidelines. In this study, a group of 60 adult volunteers (30 men, 30 women; aged 18–25 years; mean age, 20.9 ± 1.041 years) was subjected to MR imaging (1.5 T scanner, GE). Informed consent was obtained from all of them, and none of them had any history of cardiovascular, neurological, endocrine and cervical disorders.

#### Cine-PC MR imaging

A cine-PC MR imaging method^21^ was used to measure the cardiac-gated CSF flow through the transverse plane at the level of the upper border of atlas with a peripheral pulse trigger (e. g., finger photoplethysmography). Imaging parameters were collected as follows: TR, 33 ms; 10 TE, 10 ms; flip angle, 20°; imaging matrix, 256 × 192; FOV, 240 cm^2^; section thickness, 5 mm; and 2 signal intensity average. The encoding direction was in the head-to-foot orientation for all volunteers. PC-images were obtained at each time point, for a total of 25 measurements equally distributed over the cardiac cycle. The imaging time duration varied between 2 to 5 min, depending on the volunteer’s heart rate.

#### Experimental procedure

**Figure 1:**
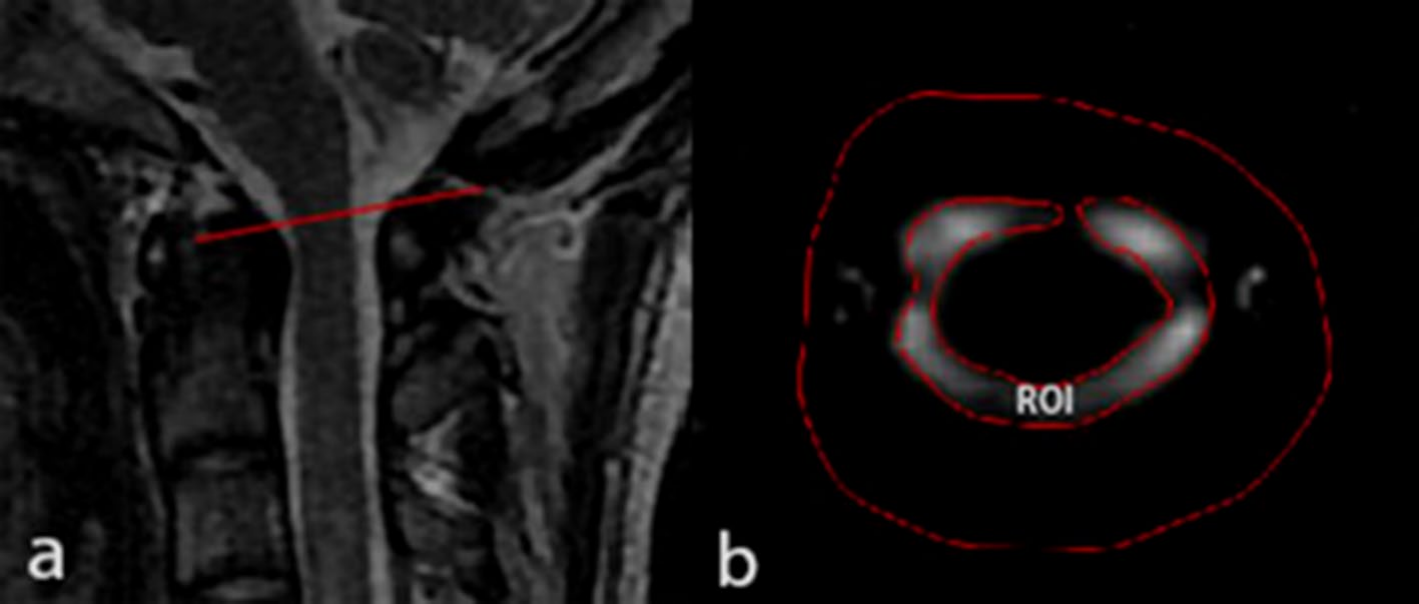
Example of MR images from one of the volunteers. The region of interest is the entire cross-sectional area of the subarachnoid space at the level of the upper border of atlas. (**a**) Showing the level of upper border of the atlas (red line) in the median sagittal plane of nape; (**b**) the region of interesting (ROI, inner closed irregular contour) showing the entire cross-sectional area of the subarachnoid space in the transverse plane at the level of the upper border of atlas. And the outer red line serves as a guideline.

Each volunteer was scanned twice. The first scanning was performed after 10-min resting period. The second scanning was performed after one-minute-head-nodding. Before the cine-PC scan, sagittal T2 weighted images of head and neck were obtained by a quick MR scan to provide anatomic details. Based on the median sagittal images, a transverse plane for the cine-PC scan was designated at the level of the upper border of atlas (Fig. 1a).

#### Head-nodding standard

Volunteers lied on an MR bed with a cushion pillow, One head-nodding cycle was counted when a head extended from the neutral position to the largest range and back to the origin and then flexed from the neutral position to the largest range and finally back to the neutral position. Volunteers nodded their heads according to the researcher’s instructions for one-minute-head-nodding normalization of 30 times within 1 min.

**Figure 2:**
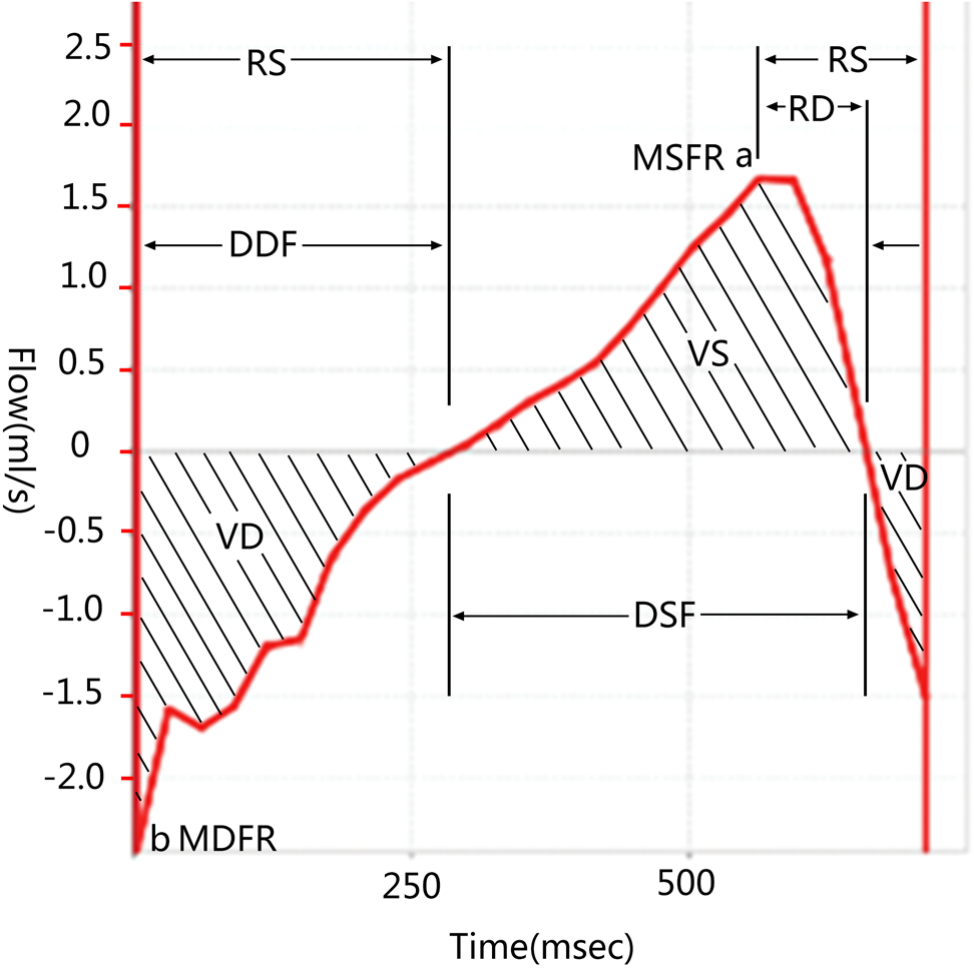
Example of a CSF flow rate waveform from one of the volunteers, with a graphical representation for most parameters analyzed. Positive waveform values correspond to systolic (craniocaudal orientation) CSF flow, while negative values correspond to diastolic (caudocranial orientation) CSF flow. *R–D* the interval ranging from a R wave to the onset of the diastolic CSF flow, *R–S* the interval ranging from a R wave to the onset of the systolic CSF flow, *DSF* duration of the CSF systolic flow, *DDF* duration of the CSF diastolic flow. *a* peak of the systolic curve, representing the maximum systolic flow rate (MSFR), *b* trough of the diastolic curve, representing the maximum diastolic flow rate (MDFR), *VS* area under a systolic curve, representing the CSF flow volume during the systole, *VD* area under a diastolic curve, representing the CSF flow volume during the diastole. In addition, some parameters obtained by calculations, the average systolic flow rate (ASFR) = VS/DSF, the average diastolic flow rate (ADFR) = VD/DDF, and the CSF stroke volume during the entire cardiac cycle (net flow volume, NV) = VS + VD.

Cine-PC images were transferred to an independent workstation (AW46MR) for CSF flow analysis using the analyzing (Report Card 4.0) software. Using the region-of-interest function of the analyzing software, an irregular contour was drawn manually to encompass the entire cross sectional area of the subarachnoid space at the level of the upper border of atlas (Fig. 1b). The region-of-interest statistics output from the analyzing software included the mean velocity values (ml/s) [(the average speed of all pixels in the ROI) × (the area of the ROI)] at each time point during the cardiac cycle. Results were plotted as waveforms with flow rate on the y-axis and cardiac cycle fractions on the x-axis (Fig. 2). On a waveform, positive values corresponded to systolic (craniocaudal orientation) CSF flow and negative values corresponded to diastolic (caudocranial orientation) CSF flow. The CSF flow rate waveforms were analyzed according to the temporal and amplitude parameters (Fig. 2)^20^.

### Measurement of CSF pressure during the lumbar puncture

#### Subjects

100 patients (79 men, 21 women; aged 17–87 years; mean age, 55.40 ± 16.95 years) without nervous system diseases or other diseases that can influence the CSF circulation and pressure, and no adverse reaction was found after the operation.

#### Materials

A disposable puncture anesthesia kit (5#1.6 × 80 mm epidural puncture needle, 16#0.5 × 110 mm nib-type lumbar anesthesia needle. Produced by Jiangsu Ruijing Science and Technology Development Co., Ltd.) and cerebral pressure measuring tube (Jiangsu Huaxing Medical Equipment Industry Co., Ltd. production) were used in this study.

#### Experimental procedure

The patients were prepared for a routine lumbar puncture, and blood pressure were monitored. Patients maintained lying on the side with their elbow, hip and knee joints flexed. The surface of back maintained vertical perpendicular, and head cushion is prepared to make sure the head, neck, and spine horizontal. The L3–L4 posterior intervertebral space was selected as a puncture point. A 5# (1.6 × 80 mm) epidural needle and a pen tip type 16# (0.5 × 110 mm) spinal needle were used. When CSF flows out, the piezometer tube was connected. When the fluid level in the piezometer tube slowly rose and stabilized, the CSF pressure (mmH_2_O), blood pressure (mmHg), and heart rate (times/min) were recorded. Subsequently patients remained in the lateral position with a cushion pillow and gently performed nodding movement 5 times. When the fluid level in the piezometer tube got stabilized again after the head-nodding movement, the above parameters were recorded again.

### Data analysis

The mean ± standard deviation or quartiles were calculated for each parameter. All parameters were compared between both measurements acquired before and after the head-nodding period. Statistical significance was calculated using a paired sample *t* test, rank sum test and Chi-square test in SPSS 17.0. *p* value of less than 0.05 indicated a statistically significant difference.

## Result

### Changes in cerebrospinal fluid flow after the head-nodding

**Figure 3:**
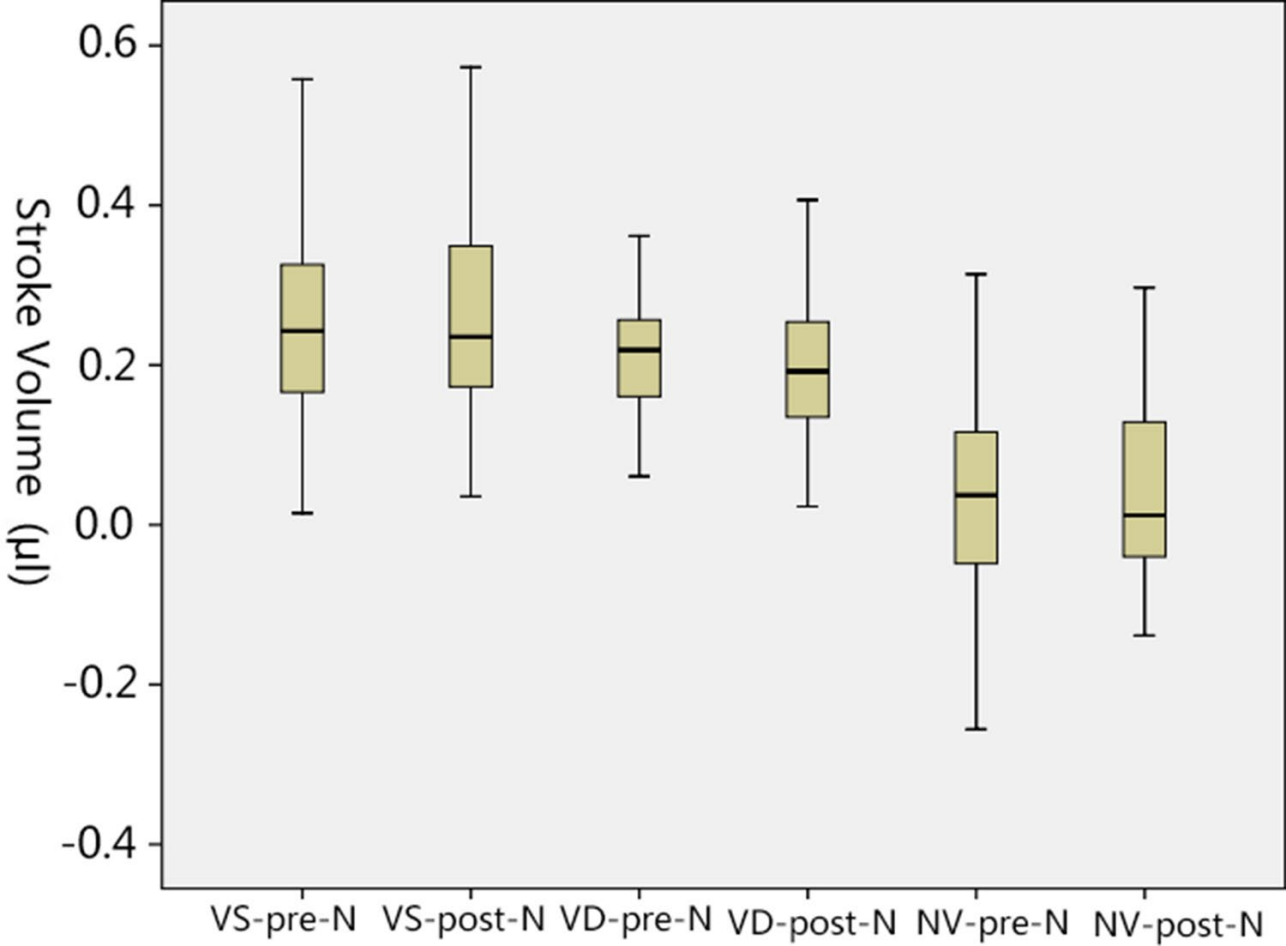
The CSF dynamics volumetric parameters were showed in Box and Whisker plots. It was impossible to re-measure the subjects with outliers, so these outliers were not included. VS-Pre-N (Pre-nodding VS) and VS-Post-N (Post-nodding VS), n = 59; VD-Pre-N (Pre-nodding VD) and VD-Post-N (Post-nodding VD), n = 56; NV-Pre-N(Pre-nodding NV) and NV-Post-N (Post-nodding NV), n = 56.

The cardiac cycle-related CSF flow pulsations were depicted as CSF flow waveforms. Based on the waveforms, the CSF flow quantitative parameters of the occipitocervical junction were calculated. And the results of both scans and the comparisons between them were presented in Tables 1, 2, 3 and 4 and Figs. 2 and 3.

**Table 1:** Comparison of the temporal parameters obtained before and after the head-nodding period (paired *t* test).

Value	R–S		DSF		DDF	
	Pre-nodding	Post-nodding	Pre-nodding	Post-nodding	Pre-nodding	Post-nodding
N	52		52		56	
Ranges	0.261–0.680	0.255–0.777	0.326–0.629	0.283–0.769	0.149–0.496	0.117–0.571
Mean ± SD	0.460 ± 0.105	0.466 ± 0.123	0.463 ± 0.073	0.489 ± 0.115	0.306 ± 0.079	0.320 ± 0.103
t value	− 0.400		− 1.702		− 1.087	
p value	0.691		0.095		0.282	

Temporal values were given in milliseconds. The outliers were removed on the base of Box and Whisker Plots analysis.

**Table 2:** Comparison of the temporal parameters obtained before and after the head-nodding period (Wilcoxon rank-sum test).

Value	R–D	
	Pre-nodding	Post-nodding
Quartiles	0.087; 0.139; 0.229	0.088; 0.138; 0.214
N	59	
z value	− 0.23^a^	
p value	0.982	

^a^Based on positive rank. Temporal values were given in milliseconds. The outliers were removed on the base of Box and Whisker Plots analysis.

**Table 3:** Comparison of the amplitude parameters obtained before and after the head-nodding period.

Value	MSFR		MDFR		ASFR		ADFR	
	Pre-nod	Post-nod	Pre-nod	Post-nod	Pre-nod	Post-nod	Pre-nod	Post-nod
N	60		56		52		50	
Ranges	0.098–2.097	0.274–2.094	0.600–2.767	0.781–2.890	0.390–1.091	0.104–1.090	0.200–1.106	0.160–1.137
Mean ± SD	0.943 ± 0.445	0.924 ± 0.393	1.965 ± 0.531	1.839 ± 0.460	0.535 ± 0.257	0.514 ± 0.230	0.702 ± 0.253	0.606 ± 0.228
t value	0.347		2.282		0.546		2.451	
*p* value	0.730		0.026		0.587		0.018	

Amplitude values were given in milliliters per second. The outliers were removed on the base of Box and Whisker Plots analysis.

**Table 4:** Comparison of the volumetric parameters acquired before and after the head-nodding period.

Value	VS		VD		NV	
	Pre-nodding	Post-nodding	Pre-nodding	Post-nodding	Pre-nodding	Post-nodding
N	59		56		56	
Ranges	0.015–0.558	0.036–0.573	0.061–0.362	0.023–0.407	− 0.256–0.314	− 0.139–0.297
Mean ± SD	0.255 ± 0.136	0.253 ± 0.124	0.212 ± 0.078	0.205 ± 0.092	0.035 ± 0.110	0.040 ± 0.113
t value	0.119		0.582		− 0.398	
*p* value	0.906		0.563		0.692	

Volumetric values are given in microliters. The positive value above means flowing caudal direction. The outliers were removed via Box and Whisker Plots analysis.

#### Temporal analysis

The temporal analysis of CSF dynamics were listed in Table 1 which compared the mean values of R–D, R–S, DSF and DDF (R–D, the interval ranging from a R wave to the onset of the diastolic CSF flow; R–S, the interval ranging from a R wave to the onset of the systolic CSF flow; DSF, duration of the CSF systolic flow; DDF, duration of the CSF diastolic flow) (Fig. 2). No statistically differences were observed between the pre- and post-head-nodding temporal parameters (Tables 1 and 2).

#### Amplitude analysis

The CSF dynamics amplitude parameters were listed in the Table 3 which compared the mean values of the MSFR, MDFR, ASFR and the ADFR (MSFR, the maximum systolic flow rate; MDFR, the maximum diastolic flow rate ; ASFR, the average systolic flow rate; ADFR, the average diastolic flow rate) (Fig. 2). The MDFR and the ADFR were significantly decreased from 1.965 ± 0.531 to 1.839 ± 0.460 ml/s and from 0.702 ± 0.253 to 0.606 ± 0.228 ml/s respectively after the one-minute-head-nodding period (p value of MDFR = 0.026; p value of ADFR = 0.018), however, the MSFR and the ASFR remained unchanged (Table 3).

#### Volumetric analysis

The volumetric analysis of CSF dynamics were listed in the Table 4 which compared the mean values of VS, VD and NV (VS, the CSF flow volume during the systole; VD, the CSF flow volume during the diastole; NV, the CSF stroke volume during the entire cardiac cycle) (Fig. 2). The value distribution of these parameters was shown in Fig. 3. No statistically differences in the volumetric parameters were observed (Table 4).

In addition, the analysis of CSF stroke volume variations after the one-minute-head-nodding period were listed in Table 5. After the one-minute head-nodding period, the NV values of 29.6% subjects were strengthened in the cranial orientation following, whereas the NV values of 70.4% subjects were strengthened in the caudal orientation in the 27 subjects with an initial caudal NV direction. The NV values of 60.6% subjects were strengthened in the cranial orientation, whereas the NV values of 9.4% subjects were strengthened in the caudal orientation in the 33 subjects with an initial cranial NV direction. The NV pattern changed significantly between both subgroups (p = 0.017).

**Table 5:** Change tendency in CSF stroke volume orientation for both initial orientation states following the one-minute head-nodding period (n = 60).

Pre-nodding	Post-nodding		Total
	Strengthened in cranial orientation	Strengthened in caudal orientation	
Caudal direction	8 (29.6%)	19 (70.4%)	27 (100%)
Cranial direction	20 (60.6%)	9 (39.4%)	33 (100%)
Total	28	32	60

Chi-square test, χ^2^ = 5.725(continuity correction), p value = 0.017.

Additionally, the intervals between the one minute-head-nodding and the second cine-PC scan ranged from 1.17 to 4.00 min, with a mean value of 2.90 ± 0.40 min. It was caused by the preliminary MRI scan before the second formal scan. During the scanning procedures, the heart rates of each subject were monitored. The mean heart rates pre- and post- head-nodding period were 71.91 ± 10.62 and 69.33 ± 8.88 beats per minute. The heart rate after the head-nodding period has decreased (p = 0.000). The respiratory rate values before and after the head-nodding period were of 15.75 ± 3.37 and 15.56 ± 3.932 times per minute, respectively, showing no significant change in respiratory rate after the head-nodding period (p = 0.509). The correlation between heart rate and MDFR was not significant (p = 0.261, correlation coefficient = 0.167), but its correlation with ADFR was significant (p = 0.043, correlation coefficient = 0.307).

### Changes in cerebrospinal fluid pressure during the head-nodding

The CSF pressures and heart rates and blood pressures before and after head-nodding period were presented in the Tables 6 and 7. The CSF pressures at the L3–L4 level were markedly increased 116.03 ± 26.13 to 124.64 ± 26.18 mmH_2_O. Significant differences were found in CSF pressure (p = 0.000), heart rate (p = 0.000) and systolic blood pressure (p = 0.000), but there was no significant difference in diastolic blood pressure (p = 0.293).

**Table 6:** Comparison of the CSF pressure, heart rate and diastolic blood pressure obtained before and after the head-nodding period (paired *t* test).

Value	CSF pressure		Heart rate		Diastolic blood pressure	
	Pre-nodding	Post-nodding	Pre-nodding	Post-nodding	Pre-nodding	Post-nodding
N	99		94		94	
Mean ± SD	116.03 ± 26.13	124.64 ± 26.18	80.87 ± 10.99	83.00 ± 10.76	81.91 ± 9.76	82.48 ± 8.75
t value	− 12.334		− 5.308		− 1.057	
p value	0.000		0.000		0.293	

CSF pressures are given in mmH_2_O. The heart rates are given in times/min. The diastolic blood pressures are given in mmHg.

**Table 7:** Comparison of systolic blood pressures before and after the head-nodding period (Wilcoxon rank-sum test).

Value	Systolic blood pressure	
	Pre-nodding	Post-nodding
Quartiles	186.50; 211.00; 234.50	130.00; 142.00; 158.00
N	93	
z	− 8.357	
p	0.000	

Systolic blood pressure was given in mmHg.

Further analysis of the correlations showed no statistically significant difference among the CSF pressures, heart rates and blood pressures before and after the head-nodding period. The p value between CSF pressures and heart rates is 0.804, and their correlation coefficient is 0.029. The p value between CSF pressures and systolic blood pressures is 0.162, their correlation coefficient is 0.160. The p value between CSF pressures and diastolic blood pressures is 0.456, their correlation coefficient is − 0.087.

## Discussion

Head movements are complicated movements involving activities of many muscles. Amongst these muscles, the rectus capitis posterior minor (RCPmi), rectus capitis posterior major (RCPma), and obliquus capitis inferior (OCI) are located deep and posterior to the axis (C1) and atlas (C2). It was well known that they were connected to the upper cervical spinal dura mater via the MDBs through the posterior atlanto-occipital and atlanto-axial interspaces^1–6^. Furthermore, studies have shown that the suboccipital musculatures including RCPmi, RCPma, OCI, and the nuchal ligament (NL) participated in forming the MDB complex^22^. The MDB fibers from different origins could associate with each other and synergistically exert a pulling effect on the dura mater^22,23^.

Sui et al.^19^ and Zheng et al.^6^ proposed that the MDB may act as a pump for CSF circulation dynamics, and Xu et al.^20^ research demonstrated that CSF diastolic flow was significantly affected by the one-minute-head-rotation period, indirectly supporting the MDB's CSF circulation dynamics hypothesis.

As a further investigation upon the function of head movements, the present study found that the CSF flow was significantly affected in the one-minute-head-nodding period and the MDFR and ADFR were significantly decreased. That means nodding-head has a strong tendency to slow down the cerebrospinal fluid pulsation velocity toward the cranial cavity, although there was no obvious impact on the volume parameters (VD, VS, NV) of CSF flow.

Furthermore, the present research found that the effect of head-nodding on CSF circulation was based on the initial NV flow direction. In 70.4% of the subjects with an initial caudal NV flow direction, the NV values were strengthened in the caudal orientation following the one-minute-head-nodding period, while in 60.6% of the subjects with an initial cranial NV flow direction, the NV values were strengthened in the cranial orientation. This indicates that the effect of head-nodding on CSF circulation may have individual differences and depend on its initial direction. For most subjects, the effect of nodding on CSF circulation may increase net flow in the same direction.

Compared with the previous studies, Xu et al.^20^ found that the MDFR and ADFR were significantly increased following the one-minute-head-rotation period. That means head-rotating has a strong tendency to speed up the cerebrospinal fluid pulsation velocity toward the cranial cavity. That is contrary to the effects of head-nodding. According to the anatomy and physiology of head movements, nodding motion happens at the atlanto-occipital joint along the coronal axis, while head rotating happens at the atlantoaxial joint along the vertical axis. They are different head movements. During nodding head, the RCPma and RCPmi assist in this movement. Their MDB may pull the upper cervical spinal dural sac along the sagittal plane, and cause the posterior wall of the dural sac to move backwards. And then, that may result in the decline of the MDFR and ADFR with some kind of kinetic mechanism. However, during head shaking, the OCI works with other muscles, such as the sternocleidomastoid, to move the atlantoaxial joint. In this process, the OCI’s MDB may pull the posterior wall of upper cervical spinal dural sac laterally and cause the dural sac to twist following the head-rotating. And then, that may lead to the increase of the MDFR and ADFR with some kind of kinetic mechanism. For the conflict between CSF flow rate and volume, we purposed it may be related to changes of the cross-sectional area at the measurement location. However, we still need to explore this conflicting finding in future study.

Accordingly, the present study also found that the CSF pressure in the lumbar region was significantly increased during 5 times head-nodding movements. Based on the results of Cine-PC MRI measurements, the cranial flow rate of CSF was slowed down during head-nodding. it means that more and more cerebrospinal fluid might be transported into the spinal canal during head-nodding and as a result, the CSF pressure would be increased of in the spinal canal. The results of CSF pressure measurement proved the speculation. Consequently, the present study suggests that head-nodding may provide driving force for the diffusion of cerebrospinal fluid from the cerebellomedullary cistern into the spinal canal.

Due to the subjects keeping stationary during the MRI scan, the CSF flow parameters were obtained not in sync with head-nodding but 1.17–4.00 min after head-nodding period. The immediate effects of head-nodding movement might be more pronounced than the measured effect because of the time lap between the head nodding occurring and the CSF measuring. In this way, the results of this study might be follow-up effects of nodding motion and motion mechanisms such as inertia might provide explanation for this effect.

In addition, at the level of the occipital cervical junction, the cerebellar tonsils are suspended in the cerebellomedullary cistern. During the head flexion and extension, the size of the space between the tonsils and the atlas (C1) will be changed. In this way, we suggest that the relative movement of the cerebellar tonsils might also be a factor affecting CSF circulation during head-nodding movement. That will be studied in the future.

Moreover, there are many factors that affect CSF circulation. The heart rate^24,25^ and respiration^26–29^ are well known to affect CSF circulation. In this study, it was found that the heart rate decreased significantly after head-nodding period, which might be related with volunteers’ adaptation of the MR working environment. According to the dynamic mechanism of CSF circulation^30^, the rhythmic beating of the arteries directly transmits the pulse pressure to the CSF in the brain and promotes the flow of CSF. This study showed that there was no significant correlation between heart rate changes and MDFR, but the heart rate was significantly correlated with ADFR. The lower heart rate could prolong the DDF, thus ADFR may be affected by the lower heart rate. In addition, some authors have shown that when the heart rate is less than 90 beats per minute, the change in heart stroke volume is very weak^31^. According to the way that heart rate influences the CSF, we may conclude that the heart rate changes in the study may not lead to CSF flow changes. In our current study, the respiratory rate remains statistically unchanged before and after one-minute-head-nodding movement. Therefore, it was reasonable to eliminate the interference of respiratory changes in the studies.

The results of this study also provide a basis for etiology and diagnosis of CSF circulation-related diseases. Several studies have found that changes of CSF circulation are tightly associated with some clinical disorders, such as type I Chiari malformation^32–34^ and syringomyelia^35–37^. Anatomy and pathologic changes of the head movements, the MDB, or the suboccipital muscles might be related with them. Additionally, Clinical studies have shown that the dysfunction of the suboccipital muscles under various kinds of pathologic status might be correlated with chronic cervical headache^38–40^. The findings of this study may be a clue to its potential pathogenesis.

In conclusion, head-nodding can significantly affect the CSF circulation and head movement is one of the important drivers of cerebrospinal fluid circulation.
